# A scoping review of the prevalence of antimicrobial-resistant pathogens and signatures in ready-to-eat street foods in Africa: implications for public health

**DOI:** 10.3389/fmicb.2025.1525564

**Published:** 2025-04-09

**Authors:** Hope Onohuean, Haron Olot, Fanny Eseohe Onohuean, Sarad Pawar Naik Bukke, Oluwamayowa S. Akinsuyi, Ayomikun Kade

**Affiliations:** ^1^Biomolecules, Metagenomics, Endocrine and Tropical Disease Research Group (BMETDREG), Kampala International University, Western Campus, Ishaka-Bushenyi, Uganda; ^2^Biopharmaceutics Unit, Department of Pharmacology and Toxicology, School of Pharmacy, Kampala International University, Ishaka-Bushenyi, Uganda; ^3^Department of Pharmaceutics and Pharmaceutical Technology, Kampala International University, Western Campus, Ishaka-Bushenyi, Uganda; ^4^Department of Microbiology and Cell Science, University of Florida, Gainesville, FL, United States; ^5^School of Life Sciences, University of Warwick, Coventry, United Kingdom

**Keywords:** prevalence, antibiotic-resistance, ready-to-eat (RTE), street-foods, Africa, public health

## Abstract

**Background and objective:**

Despite its critical role in individual and societal health, food hygiene remains underexplored. Antibiotic-resistant pathogenic bacteria in ready-to-eat (RTE) food threaten public health. This scoping review collected data on the epidemiological prevalence of RTE food-contaminated pathogens resistant to antimicrobial drugs and resistance genes in Africa.

**Method:**

Using electronic databases, such as PubMed, Scopus, and Web of Science (WoS), handpicked from references, pre-reviewed published articles were retrieved and analyzed according to the PRISMA-ScR guidelines.

**Results:**

The findings indicate 40 previewed published articles qualified for meta-synthesis in the scoping review with a population/case ratio of 11,653/5,338 (45.80%). The most frequently reported RTE foods were meat or beef/beef-soup, chicken or poultry products, salads, vegetable salads, and sandwiches, which harboured pathogens such as *E. coli, Salmonella, and Staphylococcus.* Antibiotic susceptibility tests revealed the use of 48 antibiotics to manage infections, following CLSI (Clinical and Laboratory Standards Institute) protocols. Moreover, 10 authors reported 54 resistance genes associated with pathogenic resistant bacteria. In addition, only 15 studies received funding or financial support.

**Conclusion:**

These findings from several researchers indicate that RTE street foods in African and resource-limited nations harbour enteric pathogens and are a significant concern to the public health system and reservoir of the spread of antibiotic resistance. This underscores the necessity of implementing effective control strategies to address challenges and limit the spread of resistant bacteria in RTE foods. The antimicrobial resistance surveillance system in the region is a significant concern. Notably, Africa needs to strengthen the national and international regulatory bodies and a health surveillance system on antimicrobial resistance, particularly among developing nations.

## Introduction

Ready-to-eat (RTE) foods are foods prepared for consumption, and they may be raw or cooked, served hot or chilled, and often eaten without any additional treatment or processing (Lund, 2019; Valiati et al., 2023). Most RTE foods in Africa include hamburgers, salads, macaroni salad, cooked/roasted meats, smoked fish, sandwiches, cheese, mushroom pasta, deli-style cheese, burritos, fajitas, freshly made sushi, desserts, and ice-creams such as doughnuts etc. (Lund, 2019; Valiati et al., 2023). The consumption of diverse RTE foods in public spaces has become prevalent globally. RTE foods are widely consumed in low- and middle-income countries (LMICs) because of their convenience, affordability, and palatability (Makinde et al., 2020). Given the essential significance of food in human life, it is imperative to uphold food safety to protect individuals from foodborne illnesses and other associated health risks (Mengistu and Tolera, 2020). At the same time, it could be a reservoir of antibiotic resistance (Igere et al., 2022b, 2024; Onohuean and Igere, 2022). Mainstream research has extensively focused on environmental sources (such as water and soil) and clinical surveillance as major reservoirs of bacteria antibiotic resistance (Igere et al., 2022a; Onohuean and Nwodo, 2023b; Regassa et al., 2023; Satán et al., 2023). There is sparse data on the distribution and prevalence of antibiotic resistance from RTE foods.

Furthermore, food safety involves preventing chemical, biological, pathogens, and other health hazards from contaminating food. However, improper handling and serving of RTE street food can cause contamination with pathogens such as *Salmonella, Vibrio* spp.*, Campylobacter, E. coli, Listeria monocytogenes, Toxoplasma gondii, Clostridium botulinum, Moulds* and other foodborne pathogens including *Cyclospora*, *Hepatitis* A, and *Cronobacter sakazakii* (Beshiru et al., 2019; Igere et al., 2022a; Onohuean and Igere, 2022, 2023). The use of contaminated water, inadequate hygiene, and environmental sanitation also impacts the contamination of RTE food, resulting in typhoid fever and diarrhoea infections (Igere et al., 2023; Onohuean and Nwodo, 2023a). Therefore, RTE foods prepared with contaminated ingredients, water, and in an unsanitary setting can contain these bacteria, which significantly impacts public health.

Food contaminated with antibiotic-resistant pathogenic microorganisms poses a significant risk to public health. In addition to infecting people, they serve as potential reservoirs of antimicrobial resistance, facilitating the transfer of antibiotic-resistant components to both related and unrelated bacterial species (Odu and Akano, 2012; Beshiru et al., 2020; Onohuean and Igere, 2022). This has contributed to the global increase in antibiotic resistance among foodborne bacteria and infections in recent years. Regrettably, in many low-income African nations, most food vendors lack significant monitoring or licences by appropriate agencies or organizations overseen or accredited by relevant bodies or organizations to validate these RTE food safety. Again, these RTE food vendors could have compromised their products due to the environment and methods employed, increasing the likelihood that some are infected with bacterial infections. Although critical to health and productivity, food hygiene has been significantly overlooked in African research.

Antimicrobials, including antibiotics, are medications used to treat or prevent bacterial infections in humans and animals. Globally, antimicrobial resistance (AMR) in RTE foods has impacted product safety and quality. Countries with rigorous food safety standards, such as the European Union and the United States, impose stricter limitations on the use of antimicrobial agents in food production (Walia et al., 2019; Kättström et al., 2022; Taylor et al., 2022). In LMICs, where agricultural practices frequently lack control, the unrestrained use of antibiotics in animal production facilitates the emergence of resistant bacteria that may infiltrate the food chain (Samtiya et al., 2022). Due to their limited processing and frequent consumption without additional cooking, RTE foods present an increased risk of the spread of antimicrobial-resistant bacteria among consumers. Furthermore, the use of antimicrobials is affected by AMR, which constitutes a significant global public health and developmental threat. Bacterial AMR is thought to have contributed to 4.95 million fatalities worldwide in 2019 and been directly responsible for 1.27 million deaths (Murray et al., 2022; WHO, 2023).Resistance is a natural occurrence intensified by excessive or improper use (WHO, 2018; Onohuean et al., 2022a; Somda et al., 2023). Followed by mutations, gene transfer, and target modification, antimicrobial-sensitive organisms can develop microbial resistance to antimicrobials (Muteeb et al., 2023; Onohuean and Igere, 2023). Bacteria can increase antibiotic resistance through many processes, which differ according to species and origin. Thought resistance was common among clinical strains, it has become a widespread environmental and food bacterial isolate (Somda et al., 2021); one of the biggest public health issues is global.

Research has revealed a troubling degree of antibiotic resistance in the bacterial pathogens present in these foods, potentially resulting in profound health implications for consumers. The 11 AMR genes included tetracycline (*tetA*, *tetB*, *tetM*), aminoglycoside (*aadA*, *aphA-1*), sulphonamide (*sulI*, *sulII*), chloramphenicol (*cmlA*, *floR*), erythromycin (*ermB*), and disinfection resistance genes (*qacE*). *TetA* has been reported as the most prevalently identified gene in food samples, with a frequency of 100% across all samples (Xiong et al., 2019). Also, the study by Somda et al. (2023) identified *tet(A), tet(B), tet(C), tet(K), blaTEM, catA1, catA2, cmlA, blaCTXM, qnrA, qnrB, qnrS, parC,* and *qepA4* genes as typical in foodborne pathogenic bacteria in West Africa.

The widespread prevalence of antibiotic-resistant pathogenic bacteria in RTE foods significantly contributes to human multidrug resistance. The food chain acts as a transmission route for resistant strains, facilitating the acquisition and of transfer resistance genes, which pose significant health risks. Evidence has shown that multidrug-resistant bacteria in food correlate with the growth of antibiotic-resistant illnesses in humans, thereby complicating treatment (Himanshu et al., 2022; Choy et al., 2024). This underscores the critical need for surveillance data to inform policymakers and stockholders about enhanced food safety protocols. The goal of this scoping review is to create a database for RTE food resistance to antimicrobial drugs in Africa. To identify the most incriminated pathogens, we assessed the evolution of antibiotic resistance in bacteria isolated from RTE food samples and determined the prevalence of antibiotic resistance genes in RTE food.

## Methodology

### Study design and article search strategies

This study adopted the protocol of Systematic Reviews and Meta-Analyses extension for Scoping Reviews (PRISMA-ScR) (Tricco et al., 2018) for the study selection process of pre-review published articles from African countries. Using specific keywords and wildcard* procedural details in Supplementary file. Research articles from PubMed, Scopus, and Web of Science (WoS), published in English between 2000 and 2024, were retrieved on 25 September 2024, and updated plus handpicked from references on 29 October 2024 at 11:05 p.m. The dataset was merged and normalized using the ScientoPy and fBasics R packages, duplicates were eliminated, and the final compilation was saved in CSV or Excel format (Rstudio Team, 2020; Onohuean et al., 2022b, 2022c).

### Eligibility criteria

Articles that satisfied the specified inclusion criteria were included in the systematic review. Research articles conducted within the African nations between 2000 and 2024, a defined research methodology such as cross-sectional, survey, experimental or bacteriological research, conventional phenotypic method or genotypic primary Polymerase chain reaction (PCR) methods or molecular methods for antibiotic resistance genes or whole genome sequencing and MALDI-TOF mass spectrometry to identify the prevalence of antibiotic-resistant pathogenic bacteria of public health-relevant bacteria, such as *Salmonella*, *Staphylococcus aureus*, *Vibrio* spp., and *E. coli*, and resistance genes in RTE foods, articles published in English in language, studies conducted on various types of RTE foods in Africa nations qualified for inclusion. The excluded articles were review articles, studies on antibiotic resistance genes in artificially contaminated bacteria or machine learning studies, research theses, opinion pieces, book chapters, non-peer-reviewed works, clinical or environmental sample sources that are not RTE foods, and conference abstracts for which full articles were not readily available.

### Outcome of interest

The outcome of interest based on the objective of this systematic review is to determine the prevalence of RTE food-contaminated pathogens (*E. coli*, *Salmonella*, *Staphylococcus aureus,* etc.), their resistance to antimicrobial drugs, and resistance genera within African nations.

### Assessment of data quality

The Newcastle-Ottawa Scale (NOS), approved by the Agency for Healthcare Research and Quality (AHRQ), was used to evaluate the quality of the data included in this scoping review and analysis (Onohuean et al., 2022c). Using a star rating system, the quality of the studies was evaluated based on three categories: the selection of research groups received a maximum of 4 stars, variables include articles source or type of RTE foods, representativeness sample of community or population bacteria or contaminated pathogens, antimicrobial drugs, and/or resistance-genes. The comparability of groups received a maximum of 2 stars, variables assessed include methods and period, and the assessment of outcomes received a maximum of 3 stars, variables that clearly defined and reliably measured outcomes such as the present of RTE food-contaminated pathogens, antimicrobial drugs, and resistance-genes.

### Data extraction

The two authors (HO and OH) independently analyzed the titles and abstracts of the retrieved datasets for potentially appropriate studies. Studies not conducted in Africa, as well as literature reviews, systematic reviews, conference papers, and opinion pieces, were excluded. The complete texts of the selected papers were meticulously analyzed, and the results aligned with the objectives of the scoping review, which was a meta-synthesis using a data extraction tool. The information, including title of articles, authors/year, countries, source (RTE foods), method, types of bacterial isolates, sample population, antibiotic/antimicrobial used, antibiotic susceptibility pattern, resistant genes, and funders, was obtained in the data extraction form.

### Data analysis

The retrieved data were used for descriptive statistics. Subsequent analysis was conducted at several stages in Excel 2013 R version 4.1.0 software, and findings are presented in figures, graphical heatmaps, etc.

## Results

### Summary findings from the literature search

**Figure 1:**
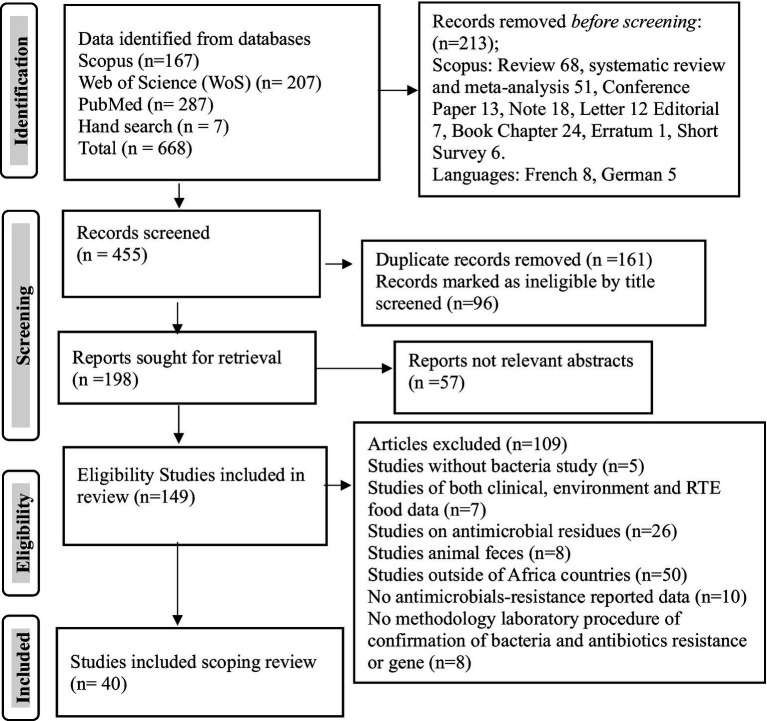
PRISMA study selection flowchart on antibiotic-resistant pathogenic bacteria, prevalence rates, and resistance genes in RTE foods in Africa.

**Figure 2:**
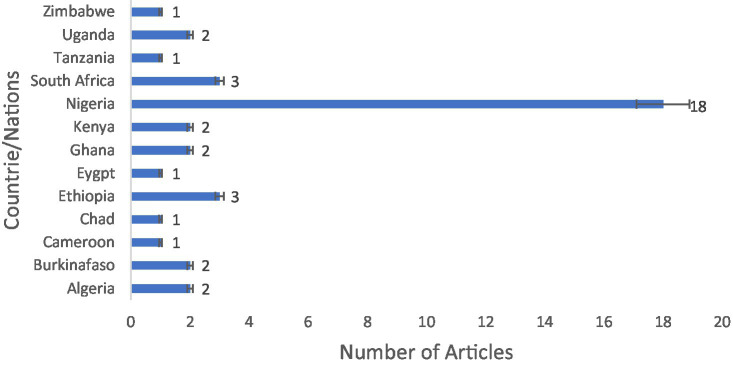
Study localities on antibiotic-resistant pathogenic bacteria, prevalence rates, and resistance genes in RTE foods in Africa.

From the total of 668 articles identified in the searched different databases, 455 passed the initial screening, 198 were retrieved, 149 were eligible, and 40 published articles qualified for inclusion in the meta-synthesis in the scoping review (Figure 1). In the African region, the nation’s Nigeria (*n* = 18 articles), Ethiopia (*n* = 3 articles), and South Africa (*n* = 3 articles) were the countries most frequently reported on the national distribution of antibiotics recovered from RTE foods (Figure 2). However, only one study has been reported on mixed West African countries (Ghana, Nigeria, Benin, and Togo).

### Quality assessment

In Supplementary Table 1, we present the particular information on the evaluation questions listed according to a domain for each article and the quality evaluation ratings of the research that were included. On the other hand, the comparability of the NOS variables did not reward any of the research papers that were evaluated with any stars because no comparative data/studies were found in the included articles. However, the quality ratings of the included studies ranged from 8 to 10. A total of 12 studies were awarded 10 points out of the potential 10 points, 18 were awarded 9 points, and 10 were awarded 8 points.

### Articles included

The articles used in this scoping review were Oladipo and Adejumobi (2010); Nyenje et al. (2012); Akinyem et al. (2013); Zige (2013); Aminu and Umeh (2014); Akinnibosun and Ojo (2015); Owoseni and Onilude (2016); Tesfaye et al. (2016); Yaici et al. (2017); Ananias and Roland (2017); Ebakota et al. (2018); Morshdy et al. (2018); Okoli et al. (2018); Tshipamba et al. (2018); Blessed (2018); Ndunguru and Ndossi (2020); Okafor-Elenwo and Imade (2020a, 2020b); Okubo et al. (2020); Asiegbu et al. (2020); Claudious et al. (2020); Fayemi et al. (2021); Izevbuwa and Okhuebor (2021); Makinde et al. (2021); Mayoré et al. (2021); Mekhloufi et al. (2021); Nikiema et al. (2021); Setsoafia Saba et al. (2021); Karikari et al. (2022); Mwove et al. (2022); Soubeiga et al. (2022); Esemu et al. (2023); Alelign et al. (2023); Ronald et al. (2023); Beshiru and Igbinosa (2023); Akinyele et al. (2024); Isic et al. (2024); Moges et al. (2024); Umar et al. (2024).

### Population/cases of antibiotic-resistant pathogenic bacteria in RTE foods in African countries

**Figure 3:**
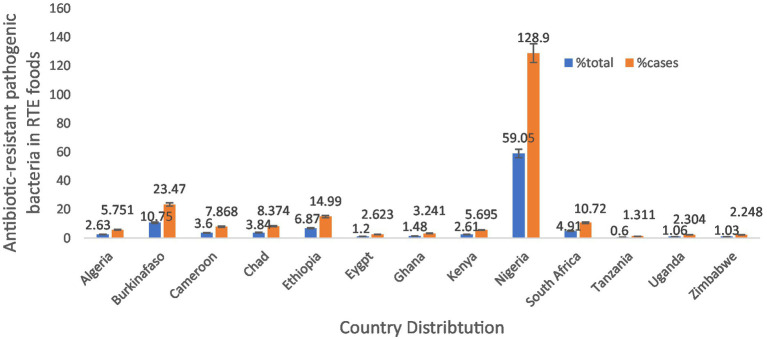
Country distribution of antibiotic-resistant pathogenic bacteria, prevalence rates in RTE foods in Africa.

In Africa, antibiotic-resistant pathogenic bacteria were found in 5,338 out of 11,653 (45.80%) samples of RTE foods. However, the sample size and cases by nations were Nigeria (59.05%, 128.9% cases), Ethiopia (6.87%, 14.99% cases), and South Africa (4.91%, 10.72% cases) were the countries most frequently reporting antibiotic-resistant bacteria from RTE foods (Table 1 and Figure 3). The, one study involving a combination of West African countries (Ghana, Nigeria, Benin, and Togo) reported fewer occurrences, accounting for only 0.37% of the total samples and 0.81% of the cases.

**Table 1:** Overall characteristics of the included studies on antibiotic-resistant pathogenic bacteria, prevalence rates, and resistance genes in RTE foods in Africa.

Authors	Countries	Source (RTE foods)	Period	Method	Types of bacterial isolates	Total samples	Positive cases	% prevalence	Antibiotic susceptibility (resistance)	Resistant genes	Funders	Score
Mekhloufi et al. (2021)	Algeria	Salads	2018 to 2019	(ISR)-PCR, (SEg)-typing	*Staphylococcus*	207	99	47.83	Benzylpenicillin	*mecA*, tst	No external funding	9
Yaici et al. (2017)	Algeria	Sandwich	February 2013 to March 2014	PCR	*E. coli, Klebsiella*	100	14	14.00	Tetracyclines, nalidixic acid, kanamycin, chloramphenicol, gentamycin, enrofloxacin, and streptomycin	*blaCTX-M-1*, *blaCTX-M-15*, *blaCTX-M-14*, *blaCTX-M-2*, *blaSHV-2*, *blaSHV12*, *blaCTX-M-1*, *blaCMY-2*, *aac(6′)-Ib-cr*, *oqxA*, *oqxB*, *q*nr*A*, *q*nr*B*, *q*nr*S*, *mecA*, *tsst-1*	French agency for food, environmental and occupational health safety (ANSES)	10
Nikiema et al. (2021)	Burkina Faso	Sandwich	June 2017 to July 2018	Whole-genome sequencing, Serotyping	*Salmonella.*	201	36	17.91	Ampicillin, ampicillin/sulbactam, amikacin, amoxicillin, amoxicillin/clavulanic acid, augumentin, azithromycin, tetracyclines, nalidixic acid, norfloxacin, mupirocin, kanamycin, ceporex, carbenicilin, chloramphenicol, gentam ycin, cefotaxime, clindamycin, and ceftazidine	*blaTEM-1B*, *tet(A)*, *Sul1*, *Sul2*, *aac(3)-IV*, *aac(3)-II*, *strA*, *strB*, *qacE∆1*, *gyrA or parC*	Cooperation and Cultural Action Service of the French Embassy of France, Burkina Faso, and Institute Pasteur and the French government’s Investissement d’Avenir Programme, Laboratoire d’Excellence ‘Integrative Biology of Emerging Infectious Diseases	10
Soubeiga et al. (2022)	Burkina Faso	Sesame, Salads-lettuce, mango-juice.	2018 to 2020	PCR	*Salmonella.*	1,052	148	14.07	Ampicillin, amoxicillin-clavulanate, cefoxitin, tetracycline, gentamicin, amikacin, chloramphenicol, trimethoprim-sulphamethoxazole and nalidixic acid	*tetA*, *tetB*, *blaTEM*, *temB*, *sul1*, *sul2*, *and aadA*	No external funding	10
Esemu et al. (2023)	Cameroon	Cake, bread, fruit salad, meat hot pot, suya, and boiled rice.	February to August 2020	PCR	*S. aureus*	420	161	38.33	Ciprofloxacin, amoxicillin, penicillin, oxacillin, erythromycin, azithromycin, clindamycin, gentamicin, and chloramphenicol	*nuc*, *mecA*	No external funding	9
Mayoré et al. (2021)	Chad	Minced beef sandwich	October 2014 and January 2018.	Disc diffusion	*Salmonella.*	447	5	1.12	Amoxicillin, Cefotaxime, Nalidixic acid.	NR	Research Project and Support for Street Food Safety (“PRASAR”)	10
Alelign et al. (2023)	Ethiopia	Sambusa, Potato Chips, Bonbolino, Koker, Ambasha	September 5th, 2022, to December 31st, 2022	Biochemical tests.	*Mixed*	330	113	34.24	Ampicillin, amoxicillin-clavulanate, cefepime, ceftriaxone, meropenem, gentamicin, azithromycin, tetracycline, doxycycline, ciprofloxacin, cotrimoxazole and chloramphenicol	NR	Arba Minch University Research Directorate	9
Tesfaye et al. (2016)	Ethiopia	Kikil, mahberawi	March to October, 2015	Bacteriological evaluation	*Salmonella.*	120	25	20.83	Ampicillin, nalidixic acid, norfloxacin, gentamicin, ciprofloxacin, streptomycin, tetracycline, kanamycin, and chloramphenicol	NR	No external funding	9
Moges et al. (2024)	Ethiopia	Samosas, eggs, and salads.	December 2022 to February 2023	Standard microbiological methods	*Staphylococcus*	350	186	53.14	Erythromycin, amoxicillin, streptomycin, ciprofloxacin, sulphamethoxazole-trimethoprim, and chloramphenicol	NR	Jimma University	9
Morshdy et al. (2018)	Egypt	Meat sandwiches	May 2017–April 2018	PCR, antibiotic susceptibility testing	*S. aureus*	140	102	72.86	Ciprofloxacin, enrofloxacin, ampicillin	*mecA*	No external funding	10
Setsoafia Saba et al. (2021)	Ghana	Fufu	November 2016 to January 2017	Kirby–Bauer disc diffusion	*E. coli and Salmonella.*	60	41	68.33	Ciprofloxacin, gentamicin, ceftriaxone, erythromycin, and ceftazidime	NR	Salary of CKSS with contribution from TP and EY	9
Karikari et al. (2022)	Ghana	Salad, fufu	July 2015 to January 2016.	Kirby–Bauer disc diffusion	*E. coli, Salmonella*	113	113	100.00	Cefotaxime, ceftriaxone, ceftazidime, gentamicin, trimethoprim/sulfamethoxazole, ampicillin, Augmentin, ciprofloxacillin, tetracycline, erythromycin, cefoxitin, cefepime, and chloramphenicol.	NR	No external funding	9
Mwove et al. (2022)	Kenya	Cereals, fruits, salads, and sausages.	September 2020 to February 2021	Standard microbiological methods	*Salmonella & Staphylococcus*	199	93	46.73	NR	NR	Germany Academic Exchange Service (DAAD)	8
Ronald et al. (2023)	Kenya	Meat	3 months	PCR	*E. coli*	105	105	100.00	Ampicillin, streptomycin amikacin, ciprofloxacin, nitrofurantoin, co-trimoxazole, tetracycline	*tetA*, *sul1*, *blaTEM*, *strA*	Centre of Excellence in Sustainable Agriculture and Agribusiness Management (CESAAM)	10
Oladipo and Adejumobi (2010)	Nigeria	Spaghetti, bean cake	January to December	Biochemical tests	*Mixed*	11	9	81.82	Amoxyllin, streptomycin, chloramphenicol, tetracycline, gentamycin, ofloxacin, augmentin, ciprofloxacin, cotrimoxazole, nitrofurantoin, ampiclox, cefroxine, and erythromycin.	NR	No external funding	8
Akinyem et al. (2013)	Nigeria	Salad, jollof rice, fried rice, beans, moimoi, dodo, white rice	NR	Bacteriological analysis	*Alcaligenes* spp. *& E. coli*	76	12	15.79	Cotrimoxazole, nalidixic acid, amoxicillin, nitrofurantoin, typhimurium, ceftazidime, cefpodoxime, and levofloxacin	NR	Faculty of Science, Lagos State University	8
Ebakota et al. (2018)	Nigeria	Salads	February to September 2015.	Disc diffusion, serial dilution, and pour plate techniques.	*L. monocytogenes*	411	90	21.90	Nitrofurantoin, gentamicin, streptomycin, ofloxacin, ciprofloxacin, pefloxacin, amoxicillin, cloxacillin, augumentin, tetracycline, erythromycin, cefuroxime, ceftazidine and ceftriazone	NR	No external funding	9
Okafor-Elenwo and Imade (2020b)	Nigeria	Vegetable salad	March 2019 to October 2019	Bacteriological analysis	*Mixed*	3,840	2,264	58.96	Cefuroxime, ampicillin, amoxicillin/clavulanic acid, ciprofloxacin, pefloxacin, ofloxacin, sparfloxacin, cotrimoxazole, gentamycin, erythromycin, and chloramphenicol	NR	No external funding	9
Aminu and Umeh (2014)	Nigeria	Meat	12 months	Disc diffusion	*Enterobacter & E. coli.*	212	115	54.25	Nitrofurantoin, ciprofloxacin, gentamicin	NR	No external funding	9
Blessed (2018)	Nigeria	Kunun-zaki	May and August, 2016	Salmonella Shigella Agar (SSA), Kirby–Bauer disc diffusion	*Mixed*	40	5	12.50	Ciprofloxacin, sparfloxacin, pefloxacin, tarivid, sparfloxacin.	NR	Ahyuwanie E. Akanet for helping out with the funds for the publication	9
Akinnibosun and Ojo (2015)	Nigeria	Salads	NR	Serial dilution and pour plate	*Mixed*	18	8	44.44	NR	NR	No external funding	8
Izevbuwa and Okhuebor (2021)	Nigeria	Rice, soup, beans.	NR	Spread plate, serial dilution	*Mixed*	30	9	30.00	NR	NR	No external funding	8
Okafor-Elenwo and Imade (2020a)	Nigeria	Fufu, salad	March 2019 to October 2019	Pour plate technique	*Coliform & Proteus, Bacillus*	640	512	80.00	NR	NR	No external funding	8
Umar et al. (2024)	Nigeria	Vegetable salad	NR	Bacteriological analysis Antibiotic sensitivity assessment	*Salmonella*	100	36	36.00	Ofloxacin, chloramphenicol, ceftazidime, cotrimoxazole, ceftriaxone, and ampicillin.	NR	No external funding	9
Akinyele et al. (2024)	Nigeria	Fura, nunu, and tuwo	NR	Antibiotic sensitivity test	*Salmonella*	3	3	100.00	Pefloxacine, ciprofloxacine, augentin, gentamycin, streptomycin, ceporex, nalidixic acid, septrin, norfloxacin	NR	No external funding	9
Isic et al. (2024)	Nigeria	Pounded yam puff-puff, okro soup	April 2021	PCR	*Staphylococcus*	400	57	14.25	Oxacillin, erythromycin, chloramphenicol, clindamycin, ceftaroline, tetracycline, gentamicin, trimethoprim-sulfamethoxazole, ciprofloxacin, vancomycin, linezolid, *gentamicin, rifampin, quinupristin-dalfopristin, oxazolidinones* and tedizolid	*aac(6′)-Ib-cr*, *q*nr*A*, *q*nr*S*, *tet(A)*, *tetC*, *tetM*, *vanA*, *vanC*, *nuc*, *mecA*, *mecC*, *tsst-1*, *ermC*, *ermA*	The Alexander von Humboldt Foundation	10
Ohunayo et al. (2024)	Nigeria	Meat	NR	Biochemical tests, antibiotic sensitivity tests	*E. coli*	100	68	68.00	Augmentin, cefuroxime, nitrofurantoin, ceftazidime, cefixime, gentamicin, ciprofloxacin and ofloxacin.	NR	No external funding	9
Zige (2013)	Nigeria	Beans porridge	NR	Biochemical differentiation	*Proteus* spp.	36	16	44.44	Ciprofloxacin, ceftazidine, cefuroxime, gentamycin, cefixime, nitrofuratoin, augmentin	NR	No external funding	8
Makinde et al. (2021)	Nigeria	Fufu, eko	NR	16S rRNA gene phylogeny, disc diffusion.	*Klebsiella and Staphylococcus*	149	99	66.44	Ampicillin cephalothin aztreonam, amoxicillin/clavulanic acid, gentamicin, tetracycline, and imipenem	NR	National Research Foundation South Africa	8
Okoli et al. (2018)	Nigeria	Roasted meat	6 months	Disc diffusion, PCR	*Staphylococcus*	255	24	9.41	Fusidic acid, cefoxitin, oxacillin, tetracycline, erythromycin, lincomycin, vancomycin mupirocin, sulfamethoxazole/trimethoprim, gentamicin, kanamycin, streptomycin, tobramycin	*tetK*, *mecA*, *tetK*, *mphC*, *ermCT*, *ermC*	No external funding	10
Fayemi et al. (2021)	Nigeria	Minced meat, suya, kilishi, roasted beef and tsire	June–August 2019	Kirby–Bauer Disc diffusion technique, PCR.	*E. coli*	180	60	33.33	Ampicillin, ciprofloxacin, enrofloxacin, nalidixic acid, norfloxacin, streptomycin, tetracycline	*stx1*, *stx2*, *eaeA*	No external funding	10
Beshiru and Igbinosa (2023)	Nigeria	Jollof rice, fish pepper soup, egusi soup	July 2021 to February 2022	PCR, Kirby–Bauer disc diffusion.	*Vibrio parahaemolyticus*	380	42	11.05	Imipenem, gentamicin, azithromycin, ampicillin/sulbactam, streptomycin, nalidixic acid, cefotaxime, ceftazidime, tetracycline, chloramphenicol, trimethoprim-sulfamethoxazole, ciprofloxacin,	*q*nr*B*, *q*nr*S*, *blaTEM*, *tet(A)*, *tet(B)*, *tetM*, *cmlA*, *dfrA*, *Sul1*, *Sul2*, *aac(3)-IV*, *aac(3)-II*, *aadA*, *intI2*, *intI1*	No external funding	10
Asiegbu et al. (2020)	South Africa	Sandwiches salads	February 2016 to August 2017	Microbiological analyses	*Mixed*	205	175	85.37	NR	NR	Research and Innovation of the University of South Africa	9
Nyenje et al. (2012)	South Africa	Vegetables, potatoes, rice, pies, beef, chicken stew.	August and November 2011	Biochemical tests, API kits.	*Mixed*	252	181	71.83	NR	NR	Govan Mbeki Research and Development Centre, University of Fort Hare	9
Tshipamba et al. (2018)	South Africa	Chicken gizzard, beef intestines	December 2015 to April 2016	Molecular and disc diffusion methods	*Mixed*	115	49	42.61	Streptomycin, ciprofloxacin, chloramphenicol, ampicillin tetracycline, and erythromycin.	NR	National Research Foundation (NRF), South Africa	9
Ndunguru and Ndossi (2020)	Tanzania	Beef soup, stiff porridge, raw vegetable salads	March and May 2019	Standard microbiological methods	*Escherichia coli*	70	47	67.14	Chloramphenicol, cefoxitin and penicillin G, carbenicillin, ciprofloxacin, clindamycin, erythromycin, gentamicin, tetracycline	NR	No external funding	9
Okubo et al. (2020)	Uganda	Meat	February and October 2018	PCR	*E. coli*	103	89	86.41	Ampicillin, cefazolin, cefotaxime, gentamicin, kanamycin, tetracycline, minocycline, nalidixic acid, ciprofloxacin, colistin, chloramphenicol, and sulfamethoxazole-trimethoprim.	*tetA*, *tetB*, *tetE*, *tetG*, *Sul1*, *Sul2*	Japan Society for the Promotion of Science KAKENHI	10
Ananias and Roland (2017)	Uganda	Chicken, beef, goat, and meat.	February 2014 to March	Laboratory analyses	*Staphylococcus & E. coli*	20	14	70.00	NR	NR	No external funding	8
Owoseni and Onilude (2016)	Mixed West Africa countries	Puff-puff (fried dough), egg rolls, buns, fried chicken, fish and meat pie, dried fish and cake	2 years	Plasmid DNA isolation, antibiotic sensitivity testing	*Mixed*	43 Ghana n = 4, Nigeria n = 29, Benin n = 5 and Togo n = 5.	43	100.00	Nitrofurantoin, augmentin, norfloxacin, tetracycline, gentamycin, ciprofloxacin, chloramphenicol, ampicillin, nalidixic acid and cefuroxime	NR	No external funding	8
Claudious et al. (2020)	Zimbabwe	Meat pies	November 2018 to April 2019	Kirby–Bauer disc diffusion	*E. coli & coliforms*	120	70	58.33	Sulphamethoxazole and gentamicin	NR	No external funding	9

### Food types/pattern, period, and study method of antibiotic-resistant pathogenic bacteria in RTE foods

The primary authors reported different RTE foods; however, the most reported RTE foods were meat or beef/beef-soup, chicken or poultry products, salads, vegetable salads, sandwiches (Table 1). A total of 32 authors reported a study period ranging from 1 month to 2 years. All the included studies reported that the method applied comprised whole-genome sequencing, conventional PCR, serotyping, bacteriological analysis, or other standard microbiological laboratory techniques (Table 1).

### Isolated antibiotic-resistant pathogenic bacteria in RTE foods

**Figure 4:**
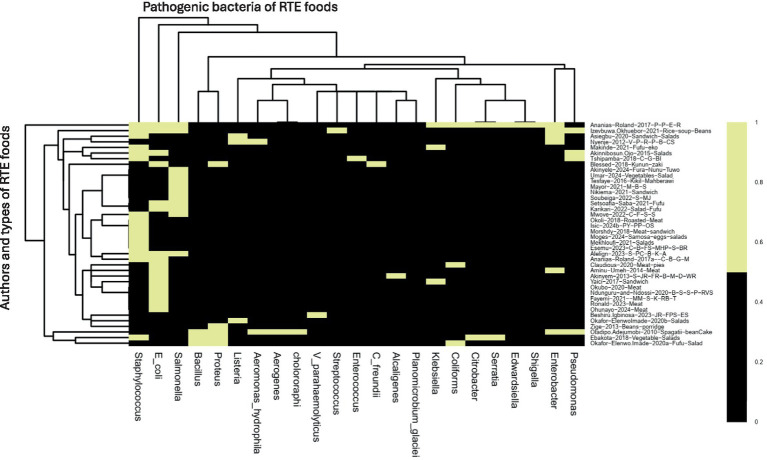
The heatmap analysis of the RTE foods samples on antibiotic-resistant pathogenic bacteria, prevalence rates, and resistance genes in RTE-foods. Black = author and antibiotic-resistant pathogenic bacteria reported. Yellow = not present. S_MJ = Salads, mango-juice. C_B_FS_MHP_S_BR = Cake, bread, fruit-salad, meat-hot-pot, suya, boiled-rice. M_B_S = Minced beef sandwich. S_PC_B_K_A = Sambusa, Potato-Chips, Bonbolino, Koker, Ambasha. C_F_S_S = Cereals, fruits, salads, sausages. S_JR_FR_B_M_D_WR = salad, jollof-rice, fried-rice, beans, moimoi, dodo, white-rice. PY_PP_OS = Pounded-Yam Puff-Puff, Okro-soup. MM_S_K_RB_T = Minced-meat, suya, kilishi, roasted-beef and tsire. JR_FPS_ES = Jollof-rice, fish-pepper-soup, Egusi-soup V_P_R_P_B_CS = Vegetables, potatoes, rice, pies, beef, chicken-stew. C_G_BI = Chicken gizzard, beef-intestines. B-S_S-P_RVS = Beef-soup, stiff-porridge, raw-vegetable-salads. C_B_G_M = chicken, beef, goat, meat. P-P_E_R = Puff-puff, egg rolls.

All the authors reported the isolated bacteria. Interestingly, 11 authors reported more than three bacterial isolates in their studies such as Yaici et al. (2017) (*E. coli*, *K. pneumoniae*, *K. oxytoca*), Alelign et al. (2023) (*Staphylococcus aureus*, *Salmonella species*, *and E. coli*), Oladipo and Adejumobi (2010) (*Bacillus licheniformis*, *Aeromonas hydrophila*, *Enterobacter aerogenes*, *Bacillus cereus*, *Proteus mirabilis*, *Pseudomonas putida*, *Proteus vulgaris*, *Pseudomonas cholororaphi and Proteus morganii*), Okafor-Elenwo and Imade (2020b) (*Serratia*, *Citrobacter*, *Proteus*, *Staphylococcus and Bacillus*, *with Proteus*), Blessed (2018) (*C. freundii*, *E. coli*, *P. mirabilis*, *P. vulgaris*), Akinnibosun and Ojo (2015) (*E. coli*, *Pseudomonas aeroginosa*, *Staphylococcus aureus*), Izevbuwa and Okhuebor (2021) (*E. coli*, *Streptococcus* spp., *Staphylococcus aureus*, *Pseudomonas aeroginosa*, *Salmonella* spp., *Enterobacterspp*), Asiegbu et al. (2020) (*Enterobacteriaceae*, *L. monocytogenes*, *S. aureus*), Tshipamba et al. (2018) (*Staphylococcus aureus S. aureus*, *Enterococcus faecalis*, *Planomicrobium glaciei*), Owoseni and Onilude (2016) (*Citrobacter*, *Edwardsiella*, *Enterobacter*, *Escherichia coli*, *Klebsiella*, *Proteus*, *Salmonella*, *Serratia*, *and Shigella*). Of all these different bacteria in any study, more than three were classified as mixed antibiotic-resistant pathogenic bacteria of public health significance. All other authors reported one or two antibiotic-resistant pathogenic bacteria in their studies, which are included in the scoping review in Figure 4 and Table 1.

### Antibiotic susceptibility patterns and resistance genes against antibiotic-resistant pathogenic bacteria in RTE foods

**Figure 5:**
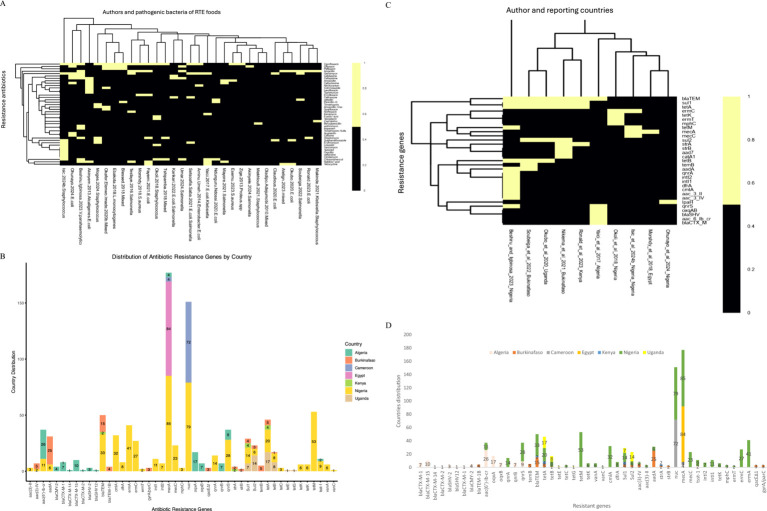
**(A)** The heatmap analysis of the susceptibility pattern on antibiotic-resistant pathogenic bacteria, prevalence rates, and resistance genes in RTE-foods. Black = intermediate/dose-dependent, and yellow = do not report any susceptibility pattern of isolates. **(B)** The countries distribution of resistance antibiotics on antibiotic-resistant pathogenic bacteria, prevalence rates, and resistance genes in RTE-foods. ampicillin/ST = ampicillin/sulbactam, amoxicillinCA = amoxicillin/clavulanic acid, QuinupristinD = Quinupristin-dalfopristin SulphametoxazoleT = Sulphametoxazole–trimethoprim. **(C)** The heatmap analysis of the resistant genes on antibiotic-resistant pathogenic bacteria, prevalence rates, and resistance genes in RTE-foods. Black = Present of the tested resistant genes, yellow = not Present. **(D)** The countries’ distribution of resistance genes on antibiotic-resistant pathogenic bacteria, prevalence rates, and resistance genes in RTE-foods.

A total of 30 authors of the included studies reported the antibiotic susceptibility patterns of 48 antibiotics used to manage pathogenic bacterial infections following the CLSI protocols. The antibiotics reported are amoxicillin, amoxicillin-clavulanic acid, ampicillin, Augmentin, azithromycin, benzylpenicillin, carbenicillin, cefepime, cefotaxime, cefoxitin, cefpodoxime, ceftaroline, ceftazidime, cefixime, ceftriaxone, cephalothin, cefuroxime, chloramphenicol, ciprofloxacin, clindamycin, cloxacillin, cotrimoxazole, doxycycline, enrofloxacin, erythromycin, fusidic acid, gentamycin, imipenem, kanamycin, levofloxacin, linezolid, nalidixic acid, nitrofurantoin, norfloxacin, ofloxacin, oxacillin, pefloxacin, penicillin G., sparfloxacin, streptomycin, sulphamethoxazole, tedizolid, teicoplanin, tetracycline, trimethoprim, trimethoprim-sulfamethoxazole, typhimurium and vancomycin. A heatmap cluster analysis highlighted the antibiotics used for bacterial isolates in each study. The two colours in the heatmap denote susceptibility patterns, such as intermediate/dose-dependent and resistance, represented with black, whereas those studies that did not report any pattern were denoted with yellow of isolates (Figure 5A). The countries’ distribution of resistance bacteria isolates to the antibiotics recovered from the RTE food among the reported studies showed Nigeria samples to have high resistance to ampicillin (4,159), cefuroxime (4,150), chloramphenicol (2,108), cotrimoxazole (2,241), gentamycin (2,302), and branded amoxicillin/clavulanic acid (1,346). Studies in Ghana showed resistance to ceftazidime (85), ceftriaxone (78), and sulphametoxazole trimethoprim (73), and ampicillin (58) isolates. Studies in Ethiopia have reported isolates resistant to erythromycin (162), ampicillin (91) and amoxicillin (68), as shown in Figure 5B.

Similarly, 10 authors reported resistance genes in seven nations in their studies, such as *aac3II, aac3IV, aac6Ibcr, aad7, aadA, blaCTXM, blaSHV, blaTEM, catA1, cmlA, dfrA, ermC, ermT, intI1, intI2, IpaH, mecA, mecC, mphC, oxqAB, qnrA, qnrS, strA, strB, sul1, sul2, temB, tetA, tetB, tetK* and *tetM* (Figure 5C). The majority of the resistance genes (*blaCTX-M-1, blaCTX-M-15, blaCTX-M-14, blaCTX-M-2, blaSHV-2, blaSHV12, blaCTX-M-1, blaCMY-2, aac(6′)-Ib-cr, oqxA, oqxB, qnrB, qnrS*) were reported in Algeria samples in Figure 5D and code for resistance to antibiotics such as beta-lactams (penicillins, cephalosporins, carbapenems), quinolones and glycopeptides (Table 2). In Nigeria, samples were observed to have several genes, namely, *qnrA, qnrS, temB, blaTEM, tet(A), tet(B), tetM, cmlA, dfrA, Sul1, Sul2, aac(3)-IV, aac(3)-II, aadA, nuc, mecA, tsst-1, intI2, intI1, tetK, mphC*, *ermT,* and *ermC* (Figure 5D) that are responsible for quinolones, penicillins, tetracyclines, chloramphenicol, aminoglycosides, sulphonamides, glycopeptides, nitrofurantoin, and macrolides (Table 2). In Burkina Faso, the studies show about seven resistance genes, *temB, blaTEM, tet(A), tet(B), Sul1, Sul2* and *aadA* (Figure 5D) and that engenders resistance to beta-lactams (penicillins, cephalosporins, and carbapenems) and sulphonamides. In Cameroon, the included studies show two prevalence resistance genes: nuc and mecA, which implies resistance to oxacillin. In Kenya, *blaTEM, tet(A), Sul1*, and *strA* are the prevalent genes accountable for resistance to beta-lactams (penicillins, cephalosporins, and carbapenems) and sulphonamides. In Uganda, studies have shown the occurrence of *tet(A), tet(B), tetE, tetG, Sul1,* and *Sul2* responsible for the resistance we find in tetracycline and sulphonamides. Egypt’s study shows sea and sec resistance genes for glycopeptides, as shown in Figure 5D and Table 2.

**Table 2:** The summary of resistance genes based on antibiotic classes in antibiotic-resistant pathogenic bacteria, prevalence rates, and resistance genes in RTE foods.

Antibiotic classes		Resistance genes
Beta-Lactams (Penicillins, Cephalosporins, Carbapenems)	*blaCTX-M-1, blaCTX-M-15, blaCTX-M-14, blaCTX-M-2, blaSHV-2, blaSHV12, blaTEM, temB*
Macrolides	*ermT, ermC, mphC*
Tetracyclines	*tet(A), tet(B), tetE, tetG, tetM, tetK*
Quinolones	*aac(6′)-Ib-cr, oqxA, oqxB, qnrA, qnrB, qnrS, gyrA and parC*
Aminoglycosides	*aac(3)-IV, aac(3)-II, aadA, strA*
Chloramphenicol	*cmlA*
Sulphonamides	*dfrA, Sul1, Sul2*
Glycopeptides	*sed, see*
Rifampin	*mecA*
Fusidic acid	*fsa*
Lincosamides	*ermT, ermC*
Miscellaneous antibiotics	
Linezolid, tedizolid	*selJ, selY, selD*
Nitrofurantoin	*intI2, intI1*
Quinupristin-dalfopristin	*selX*
Quaternary ammonium compounds (QACs)	*qacE∆1*

gyrA and parC are target mutations.

### Funders of antibiotic-resistant pathogenic bacteria in RTE foods

Only 15 authors in the included studies received funding and support for research on antibiotic-resistant pathogenic bacteria in RTE foods. Yaici et al. (2017) by the French Agency for food, environmental and Occupational Health Safety (ANSES), Nikiema et al. (2021) by Cooperation and Cultural Action Service of the French Embassy of France, Burkina Faso and Institute Pasteur and the French government’s Investissement d’Avenir Programme, Laboratoire d’Excellence ‘Integrative Biology of Emerging Infectious Diseases, Mayoré et al. (2021) by Research Project and Support for Street Food Safety (“PRASAR”), Alelign et al. (2023) by Arba Minch University Research Directorate, Moges et al. (2024) by Jimma University, Setsoafia Saba et al. (2021) by Salary of CKSS with contribution from TP and EY, Mwove et al. (2022) by Germany Academic Exchange Service (DAAD), Ronald et al. (2023) by Centre of Excellence in Sustainable Agriculture and Agribusiness Management (CESAAM), Akinyem et al. (2013) by Faculty of Science, Lagos State University, Blessed (2018) by Ahyuwanie. E. Akanet for the publication funds, Isic et al. (2024) by The Alexander von Humboldt Foundation, Makinde et al. (2021) by National Research Foundation South Africa, Asiegbu et al. (2020) by Research and Innovation of the University of South Africa, Nyenje et al. (2012) by Govan Mbeki Research and Development Centre, University of Fort Hare, Tshipamba et al. (2018) by National Research Foundation (NRF), South Africa.

## Discussion

Microbial contamination is a metric for the efficacy of food safety practices, recognized worldwide as a conduit for pathogen transmission. This scoping review indicated that all the included articles were presented with high population cases of antibiotic-resistant pathogenic bacteria in RTE food at varied degrees of contamination and are classified as unsatisfactory regarding microbiological quality. This findings from several researchers indicate that RTE street foods in the African and resource-limited nations harbour enteric pathogens and is a significant concern to public health system as well reservoir of spread of antibiotic resistance (Oladipo and Adejumobi, 2010; Nyenje et al., 2012; Akinyem et al., 2013; Zige, 2013; Aminu and Umeh, 2014; Akinnibosun and Ojo, 2015; Owoseni and Onilude, 2016; Tesfaye et al., 2016; Yaici et al., 2017; Ananias and Roland, 2017; Ebakota et al., 2018; Morshdy et al., 2018; Okoli et al., 2018; Tshipamba et al., 2018; Blessed, 2018; Ndunguru and Ndossi, 2020; Okafor-Elenwo and Imade, 2020a, 2020b; Okubo et al., 2020; Asiegbu et al., 2020; Claudious et al., 2020; Fayemi et al., 2021; Izevbuwa and Okhuebor, 2021; Makinde et al., 2021; Mayoré et al., 2021; Mekhloufi et al., 2021; Nikiema et al., 2021; Setsoafia Saba et al., 2021; Karikari et al., 2022; Mwove et al., 2022; Soubeiga et al., 2022; Esemu et al., 2023; Alelign et al., 2023; Ronald et al., 2023; Beshiru and Igbinosa, 2023; Akinyele et al., 2024; Isic et al., 2024; Moges et al., 2024; Umar et al., 2024). The notable progress in research concerning antibiotic-resistant pathogenic bacteria in RTE food could be influenced by the significance of the global threat of bacterial resistance and the emergence of infectious pathogens and outbreaks. The greater number of publication funds in Nigeria may be due to the greater number of universities and research institutions in Nigeria compared to other nations. Moreover, the level of educational attainment by the population surpasses that of other countries (Tanko et al., 2020). Other nations have very low research output on antibiotic-resistant pathogenic bacteria in RTE food, which could be elucidated by the fact that most African nations disseminate their research articles in local journals that are not listed or indexed in international databases.

This review underscores the public health risks posed by antibiotic-resistant bacteria in RTE foods, which contribute to foodborne illnesses in Africa. This specific focus on foodborne bacteria is associated with the emergence of resistance reported in environmental domains in recent years by various investigations. The bacteria present in these regions are identical to those associated with food linked to human activity, which may facilitate the development of multidrug-resistant bacteria. Different categories of food commodities have been recognized as highly consumed, as evidenced by the volume of publications concerning RTE food contamination. It can be asserted that food safety information in Africa is inadequate and disjointed, stemming from insufficient surveillance, documentation, and reporting. Furthermore, it reflects and agrees with the report of Paudyal et al. (2017) of poor or inefficient resource utilization, activity duplication, and a lack of synergy among countries in the African region. Our scoping review identified several primary categories of RTE food: chicken or poultry products, meat or beef/beef soup, and animal products and vegetables, including salads, sandwiches, and others. However, animal-derived food, such as poultry and meat products, has been widely identified as a source of illness and infections (Shange et al., 2019; Somda et al., 2023). The presence of enterobacteria in raw vegetable salads is due to their extensive distribution in soil, water, animal and human intestines, and plants. Some enterobacteria are naturally found in vegetable flora (Sagoo et al., 2003; Toe et al., 2017; Somda et al., 2023). At the same time, there are no EU or US criteria for *Enterobacteriaceae* in salad vegetables, as they naturally present in high quantities.

Epidemiological studies indicate that RTE foods are the primary sources of infectious diseases caused by *Campylobacter*, *Yersinia*, *E. coli*, *Salmonella nontyphoidal*, and several other pathogens, particularly in low-income nations in Africa. This scoping review indicated that *E. coli*, *Salmonella* spp., and *Staphylococcus* spp. were predominantly responsible for African food contamination during the study period. Most of the articles evaluated indicated that food contamination by microorganisms is associated with inadequate or violations of hygienic regulations in either handling or preparation. Moreover, pathogen-induced food contamination may be associated with the quality of raw materials, water, environment, and the existence of reservoirs and vectors in or next to food production or service locations (Igbinosa et al., 2017; Igere et al., 2022a; Onohuean and Igere, 2022; Onohuean and Nwodo, 2023a).

The public health system in the study region is generally threatened by the consumption of pathogens that contaminate RTE foods. This has been impacted by the increased use of antibiotics, particularly in populations at high risk of illness and life-threatening conditions. Aggravated by the impoverished settings of many African nations, antibiotics are indiscriminately used and sold in public spaces. All these variables significantly contribute to the proliferation of bacterial resistance to antibiotics. Furthermore, adverse economic conditions correlate with malnutrition, lack of access to safe drinking water, and poor hygiene; these are prevalent factors among populations in resource-limited countries that face an increased risk of infection and the transmission of resistant bacteria. In addition, antibiotic-resistant bacteria can infect humans through various situations: indirectly via the food chain through the ingestion of contaminated RTE foods or through direct contact with colonized or infected animals or biological materials such as blood, urine, faeces, saliva, semen, etc. (Cassini et al., 2019).

In this scoping review, *E. coli*, *Salmonella*, and *Staphylococcus* were frequently isolated resistance bacteria and were more implicated in foodborne infectious diseases, in agreement with previous findings (Adefisoye and Okoh, 2016; Igbinosa et al., 2017; Beshiru et al., 2020; Onohuean and Igere, 2022; Beshiru and Igbinosa, 2023). Several antibiotics are engaged in the management of related illnesses, thereby impacting the use of antibiotics and developing resistance to these bacteria. This indicates the overuse of antibiotics in agriculture, veterinary care, and humans. The various analyzed articles indicate the occurrence of genes including *tetA, tetB, tetC*, and *tetK*, which confer resistance to tetracycline, as well as *blaTEM*, *catA1*, *cmlA*, *blaCTXM*, and genes associated with quinolone resistance (*qnrA, qnrB, qnrS, parC,* and *qep*), similar to the findings by Igbinosa et al. (2017) and Kimera et al. (2020). However, few studies have reported resistance signatures or gene coding in isolated bacteria. This implies that analytical laboratories in many nations that were examined in this study lack sufficient technology, rendering them unable to identify specific genes associated with antibiotic resistance. Some experts assert that employing sophisticated techniques like whole genome sequencing may enhance the comprehension of the quantity, dissemination, and development of multidrug-resistant organisms.

The proliferation of resistance *CTX-M* in RTE foods is primarily attributed to human activities, as the majority of *E. coli CTX-M*, particularly the producer of the enzyme *CTX-M-15*, circulates clonally, which dominate the dominant faecal *E. coli* clones in humans, such as *ST131, ST95, ST69, ST393, ST405,* and *ST10* (Sharma et al., 2020; Aworh et al., 2021). Another potential cause of increased antibiotic resistance in RTE food may be linked to resistance genes present in sewage, water or wastewater, soil and environmental indices (Igere et al., 2020, 2024; Onohuean et al., 2021), especially genes such as [*aadA, blaOXA, blaOXA, erm (B), erm (F), mef (A), mph (E), sul1, sul3, clust, tet (39), tet (Q), tet (W)*]. Interestingly, these wastewater or sewage systems are used for the direct irrigation of crops, particularly in the urban centres of numerous African cities, and are occasionally discharged into streams and rivers that humans and animals ingest in Africa. Studies from different African nations have indicated that *Staphylococcus* isolated from various sources (pigs, pig carcasses, chicken and handler’s carcasses) carry the *mecA* gene (Igbinosa et al., 2016; Okoli et al., 2018; Ouoba et al., 2019; Mamfe et al., 2021; Somda et al., 2023). Igbinosa et al. (2016) provided a comprehensive identification of *Staphylococci* in meat, which is frequently associated with inadequate hygienic measures during slaughtering, shipping, processing, storage, and retail by those engaged in the production process. However, the *mecA* gene encodes a penicillin-binding protein (PBP2a) that is linked to a markedly reduced affinity for beta-lactams. Its presence induces methicillin resistance in Staphylococcus (Igbinosa et al., 2016; Somda et al., 2023). *qacEΔ1* is prevalent for the QAC resistance gene in Gram-negative bacteria and a deletion mutation of *qacE* (Hrovat et al., 2023).

Furthermore, the selection of antibiotics and patterns of antimicrobial consumption in some geographical locations in Africa are influenced by food, animal species, regional production patterns, types of farming systems (intensive or extensive), farming purposes (commercial, industrial, or domestic), coupled with the absence of a definitive legislative framework or policies regarding antibiotic use, as well as the size and socioeconomic status of the population, particularly among farmers, depicts the finding of the resistance genes. Resistance genes originating from agricultural environments are transferred to human diseases through lateral gene transfer.

In many African nations, antibiotics are readily available, like other over-the-counter drugs (OTC), coupled with self-prescription, resulting in overuse and abuse. Moreover, poverty compels most individuals in low-come settings to purchase cheap antibiotics to treat their ailments rather than incurring expenses from hospitals for proper diagnosis prior to antibiotic treatment. This scoping review indicates that only a limited number of antibiotics remain effective against various infections caused by pathogens. A multitude of reasons may elucidate these findings. Improper regulations for antibiotics, lack of antimicrobial awareness among the population, poor public health facilities in limited resources settings, inadequate and efficient health professionals, poor diagnostics and technological advances, self-health management, etc. The high cost of restricted antibiotics, which are typically regarded as a last resort, is used for critical cases of bacterial infections. Implementing measures to monitor the residual antibiotics that remain effective against these bacterial infections is imperative. As discussed above, the significant factors influencing AMR in Africa include the socioeconomic fallout, such as poverty, inadequate healthcare infrastructure, lack of education and awareness, availability of OTC antibiotics, urbanization, and population.

Our scoping analysis indicated that antibiotic-resistant pathogenic bacteria in RTE food research have not gained comprehensive financing and support from government entities, non-profit organizations, agencies, institutions, universities, commercial corporations, and pharmaceutical businesses. This has also impacted the progress in research and antibiotic epidemiological surveillance in poorly reported African nations. However, interrelationships among donors, funders, policymakers, and support teams are essential for progress, refining strategies, implementing initiatives, promoting stakeholder consultations, as advised by the African community, and ensuring accountability. Furthermore, it is essential to implement a proactive engagement strategy for stakeholders to mitigate the potential threat of bacterial resistance to antibiotics. The lessons from this contribution of this scoping review to the delinquency of multidrug resistance imply that the presence of antibiotic-resistant pathogenic bacteria in RTE foods considerably worsens the problem of multidrug resistance (MDR). The existence of these bacteria not only presents a direct health hazard but also enables the transmission of resistance genes to human infections, thereby complicating therapeutic alternatives. For instance, 43.5% of coagulase-negative *staphylococci* (CoNS) strains remained identified as multidrug-resistant (Chajęcka-Wierzchowska et al., 2023), and more than 95% of *E. coli* bacteria from RTE foods exhibited multidrug resistance, demonstrating elevated resistance levels to prevalent medicines such as tetracycline and ampicillin (Zhang et al., 2021; Onohuean and Igere, 2022). Of significant importance, the mobile genetic elements (MGEs) essential for the horizontal transfer of antibiotic resistance genes have been identified in *Enterococcus faecium* UC7251, which is isolated from RTE food and contains plasmids that enable the transfer of resistance genes (Belloso Daza et al., 2022). The existence of plasmid-mediated colistin resistance in *E. coli* underscores the capacity of these genes to disseminate swiftly from bacterial to human populations (Zhang et al., 2021).

AMR is associated with increased morbidity and mortality, with forecasts indicating up to 10 million fatalities per year by 2050 if current trends persist (Tang et al., 2023). Globally, AMR has become a critical global health issue, with increasing resistance to commonly used antibiotics threatening to undermine advances in medicine. Global trends in AMR reflect a rise in infections caused by resistant bacteria, driven by factors such as the overuse and misuse of antibiotics in healthcare and agriculture, poor sanitation, and inadequate infection control measures (WHO, 2023). Studies have shown that resistance is not confined to any region but is a global phenomenon affecting both high-income countries (e.g., the US and Europe) and low- and middle-income countries (LMICs) (Samtiya et al., 2022; Salam et al., 2023). In LMICs, the situation is often exacerbated by limited access to effective antibiotics, weak regulatory frameworks, and insufficient public health infrastructure (Salam et al., 2023). The RTE food industry has emerged as a significant pathway for the transmission of resistant pathogens, with studies suggesting that foodborne bacteria, particularly from animal products, can contribute to the spread of AMR (Conceição et al., 2023; Grudlewska-Buda et al., 2023; Ronald et al., 2023). Global surveillance data from WHO and the Centers for Disease Control and Prevention (CDC) indicate widespread resistance, especially to common pathogens like *E. coli*, *Salmonella*, and *Staphylococcus aureus* (WHO, 2020; Murray et al., 2022). This is compounded by the slow development of new antibiotics, creating an urgent need for more substantial prevention, monitoring, and treatment strategies across the food, healthcare, and environmental sectors. In addition, the financial ramifications of AMR are significant, with potential losses reaching trillions of dollars attributable to healthcare expenditures and diminished productivity (Ahmed et al., 2024), with a more significant consequence in low-income nations in their already poor and over-stressed medical health systems.

Going forward, a single health approach that involving an integrated interdisciplinary approach encompassing human, animal, and environmental health is crucial for efficient antimicrobial resistance management. Lessons learned from the four successful nations of Denmark, the United Kingdom (UK), the Netherlands, and Sweden on AMR mitigation strategies could strengthen and impact positive African outcomes. For instance, Denmark and Sweden’s strategy for AMR emphasized stringent rules governing antibiotic use in humans and animals (Levy, 2014; Wierup et al., 2021; Regeringskansliet, 2024; Wu, 2024). Denmark implemented policies to limit the use of antibiotics for growth promotion in animals, combined with surveillance programs to monitor antibiotic consumption and resistance patterns. The UK has an AMR policy that covers healthcare, agriculture, and the environment. The “UK Five-Year Action Plan for AMR” promotes antibiotic alternatives in agriculture, stewardship, and public awareness. This method has greatly reduced the number of human antibiotic prescriptions and associated resistance (Blake et al., 2022). Whereas, the Netherlands leverage on “One Health” approach to AMR by integrating human, animal, and environmental health sectors. By reducing antibiotic use in both human medicine and livestock, and by promoting alternative therapies, the country has reduced AMR, particularly in farm animals (Ooms et al., 2023). Therefore, stockholders in the African region must develop and implement short-, medium-, and long-term strategies that encompass one health approach. Furthermore, improving public awareness and education about AMR determinants and advocating for regulations on ethical antibiotic use are essential for addressing this problem. Other actionable recommendations for policymakers include establishing or improving national and global AMR surveillance networks to monitor AMR trends in humans, animals, and food products. Moreover, given the global nature of AMR, international cooperation is essential. Policymakers should collaborate through frameworks like the WHO’s Global Action Plan on AMR to ensure consistent policies and shared resources. Policymakers should incentivize public-private partnerships to accelerate innovation in combating resistant pathogens. Increased funding for developing vaccines, new antibiotics, alternative treatments (e.g., bacteriophages), and diagnostic tools is crucial.

### Limitations of the study

The keywords for data collection may have constrained the research focus and omitted grey literatures that does not index the databases used, which may provide unique insights or contain emerging trends, novel findings, and data that could influence the interpretation of antibiotic resistance patterns. Again, grey literature often includes unpublished studies, government reports, conference proceedings, theses, and other non-peer-reviewed sources, which may contain valuable data not indexed in traditional databases. However, we are confident that the evidence synthesized from Scopus, WoS, PubMed, and Handpicked based on a literature reference search could provide an insightful picture of the research landscape on antimicrobial-resistance pathogens and resistance genes in RTE foods in Africa.

Also, the NOS rating system is subjective, leading to different reviewers having varied interpretations of the criteria. It fails to account for all aspects of bias, such as publication bias or bias due to selective reporting. However, the NOS is a simple, transparent, and reproducible method for assessing study quality and allows for flexibility across different study designs (case–control and cohort studies).

## Conclusion

In this scoping review, some African nations were poorly represented in the antibiotic-resistant pathogenic bacteria survey following the article’s inclusion criteria and used databases. However, it increased the pathogens primarily responsible for foodborne infections, namely *E. coli*, *Salmonella*, and *Staphylococcus*. Educating food vendors and operators about proper hygiene practices can be a practical and cost-effective solution. The scoping analysis revealed that various forms of antibiotic resistance have been documented in food commodities across multiple African countries, with significant levels of resistance observed in infectious pathogens in diverse RTE foods. The regional antibiotic resistance surveillance system is a significant concern. Therefore, the use of antibiotics in agriculture, the food industry, and human health requires stringent regulations in Africa, particularly in resource-limited settings. This highlights the urgent need for effective control strategies to reduce the spread of resistant bacteria in RTE foods.

## Data Availability

The datasets presented in this study can be found in online repositories. The names of the repository/repositories and accession number(s) can be found in the article/Supplementary material.
