# Supplementary material for: A scoping review of the prevalence of antimicrobial-resistant pathogens and signatures in ready-to-eat street foods in Africa: implications for public health
Source: Front Microbiol. 2025 Apr 9;16:1525564. doi: 10.3389/fmicb.2025.1525564 (PMC12015681; doi:10.3389/fmicb.2025.1525564)
Supplement: Supplementary file 1 [file Data_Sheet_1.docx]

 Supplementary files

**Search keywords**

The general keywords used “(Prevalence OR Occurrence) AND (antimicrobial resistance) AND (resistance of bacterial) AND (foodborne pathogens OR foodborne disease) AND (drug susceptibility OR drug resistant OR resistance OR multidrug) AND (surveillance) AND the Africa”

Databases: PubMed, Scopus, and Web of Science (WoS) in

Languages: English

Year: From 2000 to 2024

From PubMed databases, articles were searched using a combination

<https://pubmed.ncbi.nlm.nih.gov/?term=%28+antibiotics*+OR+antimicriobial*+%29+AND+%28+microbial*+OR+foodborne*+OR+pathogens*+%29+AND+%28+antibiotics*+OR+antimicriobial*+%29+AND+%28+food*+%29+AND+africa+NOT+%28%28Review%5BPublication+Type%5D%29+OR+%28systematic+review%29+OR+%28meta-analysis%29%29&filter=years.2000-2024&size=200>

of Boolean logic operators (AND, OR, and NOT), Medical Subject Headings (MeSH), and keywords. The searches were done using the keywords and Boolean logic operators as following: (Prevalence∗ OR Occurrence∗ OR frequency∗ OR Contamination) AND (Microorganisms∗ OR Bacterial∗ OR Microbial∗ OR foodborne pathogens) AND (Public∗ OR Health∗ OR Public health) AND (significance∗ OR concern∗ OR hazards∗ OR risk) AND (ready to eat∗ OR street ∗ OR fast∗ OR cooked∗ OR processed∗ OR prepared∗ OR packed) AND (foods∗ OR meal∗ OR meat∗ OR fruit∗ OR fruit products ∗OR dairy product∗ OR vegetable ∗OR vegetable products, etc..) AND (Sub-Saharan ∗OR Developing∗ OR low income ∗OR middle income) AND (Countries∗ OR ∗ OR region, Africa, etc.).

Scopus

<https://www.scopus.com/results/results.uri?sort=plf-f&src=s&st1=%28+antibiotics*+OR+antimicriobial*+%29+AND+%28+microbial*+OR+foodborne*+OR+pathogens*+%29+AND+%28+antibiotics*+OR+antimicriobial*+%29+AND+%28+food*+%29+AND+africa&sid=adf11b9584e91d64eedd8629aa245126&sot=b&sdt=cl&sl=162&s=TITLE-ABS-KEY%28%28+antibiotics*+OR+antimicriobial*+%29+AND+%28+microbial*+OR+foodborne*+OR+pathogens*+%29+AND+%28+antibiotics*+OR+antimicriobial*+%29+AND+%28+food*+%29+AND+africa%29&origin=resultslist&editSaveSearch=&yearFrom=2000&yearTo=2024&sessionSearchId=adf11b9584e91d64eedd8629aa245126&limit=50&cluster=scosubtype%2C%22ar%22%2Ct%2Bscolang%2C%22English%22%2Ct>

Scopus: (Prevalence∗ OR Occurrence∗) AND (microbial* OR foodborne* OR pathogens* ) AND ( antibiotics* OR antimicrobial* ) AND (ready to eat* OR street* OR fast* OR cooked* OR processed* OR packed OR foods* OR meal* OR meat* OR fruit* OR fruit products* OR dairy food* ) AND Africa*

Web of Science,

(Microorganisms∗ OR Bacterial∗ OR Microbial∗ OR foodborne pathogens) AND (Antimicrobial Resistance∗ OR Drug susceptibility∗ OR Drug Resistant∗ OR Resistance∗ OR Multidrug Resistance∗ ) AND (ready to eat* OR street* OR fast* OR cooked* OR processed* OR packed OR foods* OR meal* OR meat* OR fruit* OR fruit products* OR dairy food* ) AND Africa

S Table 1. The Newcastle-Ottawa Scale (NOS) quality assessment of the included studies

| **Authors** | **Countries** | **Source (rte-foods)** | **Period** | **Method** | ***Types of Bacterial isolates*** | **Total sample** | **Postive cases** | **%prevalence** | **Antibiotic Susceptibility** | **Resistant_Genes** | **Score** |
| --- | --- | --- | --- | --- | --- | --- | --- | --- | --- | --- | --- |
| (Mekhloufi et al., 2021) | ⁎ | ⁎ | ⁎ | ⁎ | ⁎ | ⁎ | ⁎ | ⁎ | ⁎ | NR | 9 |
| (Yaici et al., 2017) | ⁎ | ⁎ | ⁎ | ⁎ | ⁎ | ⁎ | ⁎ | ⁎ | ⁎ | ⁎ | 10 |
| (Nikiema et al., 2021) | ⁎ | ⁎ | ⁎ | ⁎ | ⁎ | ⁎ | ⁎ | ⁎ | NR | ⁎ | 9 |
| (Soubeiga et al., 2022) | ⁎ | ⁎ | ⁎ | ⁎ | ⁎ | ⁎ | ⁎ | ⁎ | ⁎ | ⁎ | 10 |
| (Esemu et al., 2023) | ⁎ | ⁎ | ⁎ | ⁎ | ⁎ | ⁎ | ⁎ | ⁎ | ⁎ | NR | 9 |
| (Mayoré et al., 2021) | ⁎ | ⁎ | ⁎ | ⁎ | ⁎ | ⁎ | ⁎ | ⁎ | ⁎ | ⁎ | 10 |
| (Alelign et al., 2023) | ⁎ | ⁎ | ⁎ | ⁎ | ⁎ | ⁎ | ⁎ | ⁎ | ⁎ | NR | 9 |
| (Tesfaye et al., 2016) | ⁎ | ⁎ | ⁎ | ⁎ | ⁎ | ⁎ | ⁎ | ⁎ | ⁎ | NR | 9 |
| (Moges et al., 2024) | ⁎ | ⁎ | ⁎ | ⁎ | ⁎ | ⁎ | ⁎ | ⁎ | ⁎ | NR | 9 |
| (Morshdy et al., 2018) | ⁎ | ⁎ | ⁎ | ⁎ | ⁎ | ⁎ | ⁎ | ⁎ | ⁎ | ⁎ | 10 |
| (Setsoafia Saba et al., 2021) | ⁎ | ⁎ | ⁎ | ⁎ | ⁎ | ⁎ | ⁎ | ⁎ | ⁎ | NR | 9 |
| (Karikari et al., 2022) | ⁎ | ⁎ | ⁎ | ⁎ | ⁎ | ⁎ | ⁎ | ⁎ | ⁎ | NR | 9 |
| (Mwove et al., 2022) | ⁎ | ⁎ | ⁎ | ⁎ | ⁎ | ⁎ | ⁎ | ⁎ | NR | NR | 8 |
| (Ronald et al., 2023) | ⁎ | ⁎ | ⁎ | ⁎ | ⁎ | ⁎ | ⁎ | ⁎ | ⁎ | ⁎ | 10 |
| (Oladipo and Adejumobi, 2010) | ⁎ | ⁎ | ⁎ | ⁎ | ⁎ | ⁎ | ⁎ | ⁎ | NR | NR | 8 |
| (Akinyem et al., 2013) | ⁎ | ⁎ | NR | ⁎ | ⁎ | ⁎ | ⁎ | ⁎ | ⁎ | NR | 8 |
| (Ebakota et al., 2018) | ⁎ | ⁎ | ⁎ | ⁎ | ⁎ | ⁎ | ⁎ | ⁎ | ⁎ | NR | 9 |
| (Okafor-Elenwo and Imade, 2020b) | ⁎ | ⁎ | ⁎ | ⁎ | ⁎ | ⁎ | ⁎ | ⁎ | ⁎ | NR | 9 |
| (Aminu and Umeh, 2014) | ⁎ | ⁎ | ⁎ | ⁎ | ⁎ | ⁎ | ⁎ | ⁎ | ⁎ | NR | 9 |
| (Blessed, 2018) | ⁎ | ⁎ | ⁎ | ⁎ | ⁎ | ⁎ | ⁎ | ⁎ | ⁎ | NR | 9 |
| (Akinnibosun and Ojo, 2015) | ⁎ | ⁎ | ⁎ | ⁎ | ⁎ | ⁎ | ⁎ | ⁎ | NR | NR | 8 |
| (Izevbuwa and Okhuebor, 2021) | ⁎ | ⁎ | ⁎ | ⁎ | ⁎ | ⁎ | ⁎ | ⁎ | NR | NR | 8 |
| (Okafor-Elenwo and Imade, 2020a) | ⁎ | ⁎ | ⁎ | ⁎ | ⁎ | ⁎ | ⁎ | ⁎ | NR | NR | 8 |
| (Umar et al., 2024) | ⁎ | ⁎ | ⁎ | ⁎ | ⁎ | ⁎ | ⁎ | ⁎ | ⁎ | NR | 9 |
| (Akinyele et al., 2024) | ⁎ | ⁎ | NR | ⁎ | ⁎ | ⁎ | ⁎ | ⁎ | ⁎ | NR | 9 |
| (Isic et al., 2024) | ⁎ | ⁎ | ⁎ | ⁎ | ⁎ | ⁎ | ⁎ | ⁎ | ⁎ | ⁎ | 10 |
| (Ohunayo et al., 2024) | ⁎ | ⁎ | ⁎ | ⁎ | ⁎ | ⁎ | ⁎ | ⁎ | ⁎ | ⁎ | 10 |
| (Zige, 2013) | ⁎ | ⁎ | NR | ⁎ | ⁎ | ⁎ | ⁎ | ⁎ | ⁎ | NR | 8 |
| (Makinde et al., 2021) | ⁎ | ⁎ | NR | ⁎ | ⁎ | ⁎ | ⁎ | ⁎ | ⁎ | NR | 8 |
| (Okoli et al., 2018) | ⁎ | ⁎ | ⁎ | ⁎ | ⁎ | ⁎ | ⁎ | ⁎ | ⁎ | ⁎ | 10 |
| (Fayemi et al., 2021) | ⁎ | ⁎ | ⁎ | ⁎ | ⁎ | ⁎ | ⁎ | ⁎ | ⁎ | ⁎ | 10 |
| (Beshiru and Igbinosa, 2023) | ⁎ | ⁎ | ⁎ | ⁎ | ⁎ | ⁎ | ⁎ | ⁎ | ⁎ | ⁎ | 10 |
| (Asiegbu et al., 2020) | ⁎ | ⁎ | ⁎ | ⁎ | ⁎ | ⁎ | ⁎ | ⁎ | NR | NR | 9 |
| (Nyenje et al., 2012) | ⁎ | ⁎ | ⁎ | ⁎ | ⁎ | ⁎ | ⁎ | ⁎ | NR | NR | 9 |
| (Tshipamba et al., 2018) | ⁎ | ⁎ | ⁎ | ⁎ | ⁎ | ⁎ | ⁎ | ⁎ | ⁎ | NR | 9 |
| (Ndunguru and Ndossi, 2020) | ⁎ | ⁎ | ⁎ | ⁎ | ⁎ | ⁎ | ⁎ | ⁎ | ⁎ | NR | 9 |
| (Okubo et al., 2020) | ⁎ | ⁎ | ⁎ | ⁎ | ⁎ | ⁎ | ⁎ | ⁎ | ⁎ | ⁎ | 10 |
| (Ananias and Roland, 2017) | ⁎ | ⁎ | ⁎ | ⁎ | ⁎ | ⁎ | ⁎ | ⁎ | NR | NR | 8 |
| (Ananias and Roland, 2017) | ⁎ | ⁎ | ⁎ | ⁎ | ⁎ | ⁎ | ⁎ | ⁎ | NR | NR | 8 |
| (Claudious et al., 2020) | ⁎ | ⁎ | ⁎ | ⁎ | ⁎ | ⁎ | ⁎ | ⁎ | ⁎ | NR | 9 |

Nr = Not reported
